# Associations Between Autism Symptomatology, Alexithymia, Trait Emotional Intelligence, and Adjustment to College

**DOI:** 10.3389/fpsyg.2022.813450

**Published:** 2022-04-28

**Authors:** Denise Davidson, Dakota Morales

**Affiliations:** Department of Psychology, Loyola University Chicago, Chicago, IL, United States

**Keywords:** autism, alexithymia, emotional intelligence, college adjustment, emotion processing

## Abstract

It has been asserted that the socio-emotional challenges associated with autism spectrum disorder (ASD) may be explained, in part, by the higher rates of alexithymia in individuals with autism. Alexithymia refers to difficulties in identifying one’s own emotional states and describing those states to others. Thus, one goal of the present study was to examine levels of alexithymia in relation to ASD symptomatology and trait emotion intelligence (EI). Trait EI is a multifaceted concept that captures emotional competencies and behavioral dispositions A second goal was to assess whether alexithymia, ASD symptomatology and trait EI served as significant predictors of adjustment to college, including academic, social, and personal-emotional adjustment. In addition to keeping with the spectrum nature of autism, our research strategy allowed us to capture those students who may not have received a formal diagnosis of ASD but report symptoms that can be indicative of ASD. This includes women, who are less likely to receive a diagnosis of ASD even when ASD symptomatology is present. The results of the study showed that students reporting higher levels of ASD symptomatology also reported significantly higher levels of alexithymia and lower trait emotional intelligence (trait EI) than those with less or no symptomatology. Alexithymia was also negatively related to trait EI, and both alexithymia and ASD symptomatology were found to be significant predictors of trait EI. However, only trait EI was a significant predictor of adjustment to college and only for social adjustment. These findings suggest that support programs that develop trait EI skills may improve the college experience for students with ASD, regardless of alexithymia or ASD symptomatology.

## Introduction

Alexithymia is a multifaceted personality construct originally introduced by Nemiah et al. (1976) to describe patients who experience difficulties in identifying their own emotions due to impoverished representations of their emotional states. Put simply, alexithymia consists of an inability to identify one’s own emotional states and describe those emotional states to others. An important expansion on the definition of alexithymia to include the effects on interpersonal relationships was put forth by Bagby and Taylor (1997), suggesting that without knowledge of their own emotional experiences, individuals with alexithymia may not be able to readily imagine themselves in another person’s situation and therefore, can appear unempathetic and ineffective in modulating the emotional states of others.

### Alexithymia and Autism

Research has shown that alexithymia may underlie medical, psychiatric, and behavioral problems that are affected by difficulties with affect regulation, including autism spectrum disorder (Lumley et al., 2007). Autism spectrum disorder (ASD) is a neurodevelopmental (neurodiverse) disorder characterized by social-emotional difficulties and restrictive and repetitive interests and behaviors (American Psychiatric Association [APA], 2013). In individuals with ASD, the prevalence of alexithymia has been reported to be between 40 and 65%. In contrast, in neurotypical (NT) populations the prevalence ranges from 10 to 13% (Taylor et al., 1997; Salminen et al., 1999; Hill et al., 2004; Berthoz and Hill, 2005).

It has been suggested that the socio-emotional difficulties associated with ASD may be the result of co-occurring alexithymia (Bird and Cook, 2013; Kinnaird et al., 2019). In fact, it has been argued that the socio-emotional challenges associated with ASD may be better explained by the higher rates of alexithymia in this population, rather than autism *per se* (Bird and Cook, 2013). Providing support for this assertion, Cook et al. (2013) found that alexithymia, but not ASD traits, predicted reduced emotion facial recognition when participants were asked to label emotions portrayed on crossed-morphed faces. In other research, Kätsyri et al. (2008) found that individuals with autism were less accurate in labeling strongly degraded images of facial emotions when compared to neurotypical controls. However, this effect was no longer significant when alexithymia was used as a covariate in their study. Finally, research has shown that alexithymia, and not ASD, predicted difficulties in both facial and vocal emotion recognition (Heaton et al., 2012; Allen et al., 2013).

However, not all studies have shown this pattern of findings. For example, Stephenson et al. (2019) found that ASD symptomatology, but not alexithymia, predicted reduced eye fixation in an emotion identification task with dynamic and static stimuli requiring identification of targeted emotions. Moreover, others have rightly noted that while alexithymia rates in individuals with ASD may be elevated in comparison to their neurotypical peers, “alexithymia is neither necessary nor sufficient for an autism diagnosis, nor is it universal among autistic individuals” (Bird and Cook, 2013, p. 724). Finally, high rates of alexithymia have been found in individuals without ASD but with other disorders (e.g., substance abuse, addictive behaviors). In fact, alexithymia is considered a transdiagnostic disorder because it is a common deficit in disorders besides ASD (Grynberg et al., 2012).

### Trait Emotional Intelligence and Autism

Such disparate findings across emotion studies have led some to speculate that the type of tasks used to assess the independent contributions of autism and alexithymia to emotion processing skills can affect outcomes (Kinnaird et al., 2019). In the present study, we examined whether alexithymia, or ASD traits, served as a better predictor of trait emotional intelligence (trait EI). Trait EI was targeted for several reasons. Firstly, trait EI is a multifaceted concept of emotion processing that is believed to capture emotional competencies and behavioral dispositions (Mikolajczak et al., 2006). According to Petrides et al. (2007a) and others (e.g., O’Connor et al., 2019), these emotional competencies span four areas: sociability (emotion management, assertiveness, social awareness), emotionality (emotion perception, empathy, emotion expression), self-control (emotion regulation, impulsiveness, stress management) and wellbeing (trait optimism, trait happiness, self-esteem). Secondly, past research has shown robust relations between trait EI and personality (e.g., Newsome et al., 2000), social competency (Mavroveli et al., 2007), perspective-taking in social situations (Schutte et al., 2001), and psychological adjustment (Engelberg and Sjoberg, 2004; Chapman and Hayslip, 2005). Relatedly, trait EI has been shown to predict social network quality, loneliness, depression, and life satisfaction (e.g., Laborde et al., 2014; Andrei et al., 2015). Thirdly, research has shown that programs designed to improve trait EI can be successful, producing beneficial outcomes in work (management skills) and school (teacher training) settings, as well as improvements in the quality of relationships (see Kotsou et al., 2019, for a review). Finally, while trait EI is related to alexithymia, it appears to be a distinct construct. For example, Mikolajczak et al. (2006) found that trait EI was a significant predictor of both psychological and somatic symptoms over and above alexithymia.

Although trait EI has been studied infrequently in ASD, Gökçen et al. (2014) found that scores on the Autism Quotient (AQ; Baron-Cohen et al., 2001) were negatively correlated with global trait EI. AQ scores were also negatively correlated with wellbeing, emotionality, sociability and empathy factors assessed on the trait EI measure they administered. In both adolescents and adults with ASD, trait EI has also been shown to predict interpersonal relationship scores and social stress (e.g., Montgomery et al., 2012). However, research has found that “trait” and “ability” EI skills differ in ASD. Ability EI can be defined as the interrelated set of cognitive abilities and skills that include recognizing emotions and the complex relations between emotions, reasoning and problem solving (Mayer et al., 2000; Petrides and Furnham, 2000; Brackett and Mayer, 2003; Lopes et al., 2003). Ability EI reflects how individuals think and reason about social situations, whereas trait EI provides insight about how to *apply* that knowledge in socio-emotional situations. Importantly, Montgomery et al. (2010) found that ability EI did not differ in individuals with and without ASD (although see Boily et al., 2017). They asserted that when provided with enough time to reason through information, high-functioning individuals with ASD are not impaired in the cognitive processes involved in decoding emotional situations. However, when needing to apply that reasoning in social situations, they may struggle to do so. According to Montgomery et al. (2012), individuals with ASD “have the knowledge and cognition to understand and reason about emotional information, but their application (of it) in natural settings is impaired” (p. 9).

### Alexithymia, Trait Emotional Intelligence, and Adjustment to College

Such assertions support findings showing that knowledge and understanding of emotions are not significantly impaired in ASD (Harms et al., 2010), but that the application of that knowledge in real-time socio-emotional situations may be a challenge. In college settings, this may be particularly important as students are not only learning how to navigate the academic demands of college, but the social and emotional ones as well. Past research has shown that students with ASD traits often perform at levels comparable to neurotypical students academically (e.g., Jackson et al., 2018). Nevertheless, students with ASD are considerably more likely than both neurotypical students and students with other disabilities to drop out of college (Sanford et al., 2011; Taylor and Seltzer, 2011; Shattuck et al., 2012). In fact, students with ASD often report that their academic efforts and abilities will enable them to succeed (e.g., Jackson et al., 2018). In contrast, studies have shown that students with ASD exhibit less social and personal-emotional adjustment to college, feeling that they are not fully integrated into the social milieu of school (Trevisan and Birmingham, 2016). They also report heighted levels of anxiety and other personal-emotional adjustment issues (Accardo, 2017; Cox et al., 2017; Davidson et al., 2021). These socio-emotional challenges are believed to lead to feelings of disconnect in the college setting.

However, while alexithymia, ASD symptomatology and trait EI may be associated with socio-emotional issues, to our knowledge they have not been explored as potential underlying factors to college adjustment issues. Without this knowledge, it is difficult to design support programs for students with ASD that address the factors leading to socio-emotional challenges at college.

### The Present Study

In light of the studies cited, a primary goal of the present study was to examine whether alexithymia and ASD symptomatology uniquely predict trait EI. Also of interest was the extent to which alexithymia, ASD symptomatology and trait EI are associated with academic and socio-emotional adjustment to college in students with varying or no ASD symptomatology. In keeping with the spectrum nature of ASD, ASD symptomatology was treated as a continuous variable, given that ASD traits fall along a continuum in the general population (Constantino and Todd, 2005; American Psychiatric Association [APA], 2013). This allowed us to include students that exhibit ASD symptomatology without a formal diagnosis, and differs from studies that have focused almost exclusively on students with a formal ASD diagnosis recruited through their campus student accessibility offices. Such practices may exclude students, given that students with ASD are known to under-identify with these offices (e.g., White et al., 2011). Additionally, studies have shown that students may exhibit ASD traits without a formal diagnosis, especially high-functioning individuals (e.g., Newman et al., 2009; Cox et al., 2017). This is particularly true for females who are more likely than males to be under-diagnosed (Lai et al., 2019; Hull et al., 2020). Thus, women are often under-represented in studies on autism, particularly those studies with high-functioning individuals (Hull et al., 2020). Others have used a similar strategy to examine how symptoms associated with ASD impact functioning, regardless of diagnosis (e.g., Trevisan and Birmingham, 2016; Dijkhuis et al., 2020; Lei et al., 2020). With this information in mind, the following research questions were addressed:

1. What are the relations between ASD symptomatology and alexithymia, trait EI and adjustment to college? Do adults with higher levels of ASD symptomatology present higher levels of alexithymia and lower levels of trait EI than those reporting less or no symptomatology? How is ASD symptomatology related to college adjustment variables? Additionally, do the patterns of relations change for the DSM-5 compatible subscale scores (i.e., the *social communication and interaction* and the *restricted interests and repetitive behaviors* subscales on the SRS-2) on these study variables?
2. How does alexithymia relate to trait EI and college adjustment? Does a negative relation exist between alexithymia and trait EI? That is, are higher levels of alexithymia associated with lower levels of trait EI? Is alexithymia related to negative adjustment to college (academic, social, personal-emotional)?
3. Which is the better predictor of trait EI, ASD symptomatology or alexithymia?
4. To what extent does ASD symptomatology, alexithymia and trait EI predict adjustment to college?

## Materials and Methods

### Participants

A total of 150 college students at a private university in a large city in the Midwest region of the U.S. participated (*M*_*age*_ = 20;03, range = 18;04–25;02). Students were predominately (67%) Caucasian and female (82%). These demographics are consistent with the demographics of the school and the introductory psychology classes in which students were recruited. A *post hoc* power analysis was conducted to establish whether our study had an adequate sample size to determine at least a medium effect (Cohen’s *d* = 0.5) at an alpha level of 0.05. The results revealed the study had sufficient power for our analyses (Power = 0.83). Table 1 provides additional information about our sample. Note that because individuals reporting higher levels of ASD symptomatology could differ in important ways from those reporting less or no ASD symptomatology, we also provide additional information about the students in Table 1.

**TABLE 1:** Participant characteristics.

	Students ≤ *T*59 SRS-2	Students ≥ *T*60 SRS-2	*t/*χ^2^	*p*	*d/phi*
	*M* (*SD*)/*n* (%)	*M* (*SD*)/*n* (%)			
Chronological age (year; months)	19;08 (4;04)	19;10 (6;03)	1.56	0.12	0.27
Male:female	11:78	17:44	2.92	0.08	0.14
Racial/ethnic identity			14.32	0.01	0.31
White or Caucasian	65 (73%)	40 (61%)			
Black or African American	0 (0%)	1 (2%)			
Asian or Asian American	12 (14%)	14 (23%)			
Hispanic or Latino/Latina	10 (11%)	4 (6%)			
More than one race	1 (1%)	4 (6%)			
Other	1 (1%)	1 (2%)			
Year in school			7.38	0.11	0.22
Freshman	60 (67%)	40 (66%)			
Sophomore	24 (27%)	19 (31%)			
Junior	4 (5%)	2 (3%)			
Senior	1 (1%)	0 (0%)			
GPA range			1.55	0.93	0.11
3.80–4.00	18 (20%)	11 (18%)			
3.60–3.79	21 (24%)	20 (33%)			
3.40–3.59	22 (25%)	10 (16%)			
3.00–3.39	18 (20%)	11 (18%)			
2.75–2.99	6 (7%)	7 (12%)			
2.00–2.74	4 (5%)	2 (4%)			
SRS-2 *T*-score	51.3 (4.89)	66.1 (4.41)	–18.93	< 0.001	3.22
Normal (59 or lower)	89 (100%)	0 (0%)			
Mild (60–65)	0 (0%)	32 (52%)			
Moderate (66–75)	0 (0%)	27 (45%)			
Severe (76 or higher)	0 (0%)	2 (3%)			
SRS-2 raw score	44.1 (13.9)	85.9 (12.5)	–18.83	< 0.001	2.64
Alexithymia (TAS-20)					
Total	40.7 (9.7)	58.1 (8.61	–11.03	< 0.001	5.50
DIF	12.9 (4.6)	20.5 (4.9)	–9.33	<0.001	6.39
DDF	10.4 (3.5)	15.7 (3.6)	–9.05	<0.001	2.08
EOT	17.5 (4.2)	21.9 (3.9)	–6.26	<0.001	4.00
Trait emotional intelligence (TEIQue-SF)					
Global score	155.2 (19.2)	128.9 (18.1)	–8.46	< 0.001	5.80
Adjustment to college (SACQ)					
Academic	32.6 (4.7)	33.1 (6.5)	–0.63	0.53	0.15
Social	36.8 (5.8)	34.8 (4.6)	–2.25	0.03	0.80
Personal-emotional	41.5 (9.6)	37.4 (6.3)	–2.82	0.001	0.80

*Effect sizes given by Cohen’s d or phi, as appropriate. GPA, Grade point average; SRS-2, Social Responsiveness Scale, Second Edition; Results for the treatment subscales and for the overall total score of the SRS-2 are reported as T-scores (M = 50, SD = 10), with a T-score of 60 or greater indicative of the clinically significant difficulties associated with ASD and quantifies their severity. TAS-20, Toronto Alexithymia Scale; DIF, difficulty identifying feelings; DDF, difficulty describing feelings; EOT, externally oriented thinking; TEIQue-SF, Trait Emotional Intelligence Questionnaire-Short Form-total score; SACQ, Student Adaptation to College Questionnaire.*

The Social Responsiveness Scale (SRS-2; Constantino and Gruber, 2012) and students’ ASD diagnostic history, if provided, were used to determine ASD symptomatology. The SRS-2 is a widely used measure that was chosen because it has been shown to capture symptomatology that can be indicative of ASD (Chan et al., 2017). Specifically, the SRS-2 is a 65-item questionnaire that identifies the presence and severity of social impairment associated with ASD, as well as repetitive and restricted behaviors (Constantino and Gruber, 2012). On the SRS-2, individuals rate items about their behaviors during the past 6 months using a Likert-type scale ranging from 1 (*not true*) to 4 (*almost always true*). Scores on the SRS-2 are presented as *T-*scores (μ = 50, *SD* = 10) and take into account respondents’ gender, with *T*-scores of 60 and above indicative of clinically significant difficulties associated with ASD. In our sample, 89 students scored below threshold for ASD symptomatology on the SRS-2 (*M*_*age*_ = 20;05, range = 18;10–23;07) and 62 students scored above threshold for symptomatology that could be indicative of ASD on the SRS-2 (*M*_*age*_ = 20;01, range = 18;04–25;02). On the SRS-2, severity of symptoms can be further delineated, with scores of 60*T*–65*T* in the mild range, 66*T*–75*T* in the moderate range and 76*T* or higher in the severe range (Constantino and Gruber, 2012). *T*-scores in the range of 59 and below are generally not associated with clinically significant ASD symptomatology. Table 1 presents the percentage of students in our study falling within those ranges and participant characteristics of those scoring above and below the cut-off.

The SRS-2 was used because its scores corroborate gold-standard diagnostic tools of ASD (e.g., ADOS-2 and ADI-R) and the constructs that are considered essential for an ASD diagnosis (Bölte et al., 2008; Reszka et al., 2014). Specifically, two components of the SRS-2 have been validated (i.e., *social communication and interaction* and *restricted interests and repetitive behavior*) to corroborate ASD diagnostic criteria outlined by the *Diagnostic and Statistical Manual of Mental Disorders* (DSM-5; American Psychiatric Association [APA], 2013; Frazier et al., 2014).

The strategy of using the SRS-2 to examine ASD symptomatology regardless of formal ASD diagnostic history, has been used by others, including when assessing the connections between ASD symptomatology and mood disorders (Morie et al., 2019). Although not without limitations, this method has become increasingly common when working with samples with high-functioning females and with on-line samples, where observational confirmation of a diagnosis is impossible. In our sample, the SRS-2 had good internal consistency (α = 0.78).

With that said, 13 students (9 males, 4 females) indicated that they had received an ASD diagnosis and were receiving services for an autism diagnosis at the university’s student accessibility office. Although analyses were underpowered to detect significant differences between those with and without a formal diagnosis of ASD, the scatterplots of the 13 participants were compared to the larger sample. The responses of those with a previous ASD diagnosis aligned with the distribution of the larger sample across all measures and outliers were not detected. Of the students scoring below threshold for ASD symptomatology (≤*T*59), none reported that they had been diagnosed with ASD.

### Measures

#### Alexithymia

The Toronto Alexithymia Scale (TAS; Bagby et al., 1994) is a widely used 20-item self-report measure. Participants rate statements on a 5-point scale, with 1 (*strongly disagree*) to 5 (*strongly agree*). The TAS-20 consists of three subscales: difficulty identifying feelings (DIF), difficulty describing feeling (DDF) and externally oriented thinking (EOT). Sample items include: “When I am upset, I don’t know if I am sad, frightened or angry.” (DIF), “It is difficult for me to find the right words for my feelings.” (DDF), and “I prefer talking to people about their daily activities rather than their feelings.” (EOT). In our sample, the total score on the TAS-20 was found to have adequate internal consistency (α = 0.76).

#### Trait Emotional Intelligence

The Trait Emotional Intelligence Questionnaire-Short Form (TEIQue-SF; Petrides, 2009) is a 30-item measure based on EI theory, which conceptualizes EI as a personality trait. The TEIQue-SF provides a total, or global, trait EI score, along with scores on four trait EI factors: wellbeing (e.g., trait happiness and self-esteem), self-control (e.g., emotion regulation, impulsiveness), emotionality (e.g., trait empathy), and sociability (e.g., emotion management, assertiveness). Two items from each of the 15 facets of the 153-item long version of the TEIQue are included on the short form, based primarily on their correlations with the corresponding total facet scores (Cooper and Petrides, 2010). In our sample, the TEIQue-SF was found to have good internal consistency (α = 0.86).

#### Adjustment to College

The Student Adaption to College Questionnaire (SACQ; Baker and Siryk, 1999) is a 67-item self-report measure that assesses students’ adjustment to college. On this measure, students rate items using a 9-point scale, ranging from 1 (*doesn’t apply to me at all*) to 9 (*applies very closely to me*) to determine adjustment across four subscales (academic, social, personal-emotional, institutional affiliation). Although the scale provides a full-scale score, most studies focus on adjustment as measured by its four subscales. These include *academic adjustment* assesses how well the student manages the academic demands of school including the adequacy of their studying and academic efforts, as well as their attitudes toward their course of study. *Social adjustment* captures the degree to which the student has integrated themselves into the social milieu of college whereas *personal-emotional adjustment* reflects students’ psychological and physical wellbeing. Finally, *institutional attachment* captures how much a student identifies with and is emotionally attached to their university. Although scores on institutional attachment were gathered, they were not analyzed for the present study.

The SACQ was selected because it is one of the most widely used measures of college adjustment and has been well-validated, with the four SACQ domains associated with grade point average, use of campus services, and attrition (Beyers and Goossens, 2002; Credé and Niehorster, 2012). Moreover, the measure has been used previously in college students with clinical and subclinical levels of ASD symptomatology (Trevisan and Birmingham, 2016; White et al., 2016). On the SACQ, raw scores are converted to T-scores (μ = 50, *SD* = 10). T-scores are continuous and take into account year in school and sex of student. In our sample, internal consistency was found to be adequate on the subscales: academic (α = 0.77), social (α = 0.71), and personal-emotional adjustment (α = 0.79).

### Procedure

Following IRB approval and informed consent, students completed the randomized measures through a link to a secure online platform. When done, all participants were debriefed about the study.

## Results

### Preliminary Analyses

All data analyses were performed using IBM SPSS (v. 27.0; Chicago, IL, United States). Preliminary analyses were conducted on the dependent variables to ensure appropriateness of parametric procedures. This included checks for the normality of distributions and for outliers. Outliers were defined as values that were ≥ three standard deviations from the mean and were not part of the normal distribution (Cohen et al., 2003). Skewness was defined as variable values greater than ± 2.0 whereas kurtosis was defined by values greater than ± 7.0 (West et al., 1995). Moreover, the normality of the cumulative distribution was examined on each of the dependent variables (alexithymia, trait EI, college adjustment variables and SRS-2). These checks showed normally distributed data for all dependent variables in the sample and the groups in Table 1 (skewness ranged from –1.46 to 1.70; kurtosis ranged from –2.10 to 2.49) and non-significant Levene’s tests of homogeneity of variance between groups in Table 1 [*F*(1, 46) ≤ 2.57, *p* ≥ 0.11].

All analyses of total and subscale scores maintained the continuous nature of the variables. In addition, raw scores from each measure were used for all data analyses. Effect sizes and covariance inflation factor (VIF) were also calculated. VIF measures how much the variance of an independent variable is influenced or inflated by its correlation with the other independent variables and is important to determine when using regression analyses. VIF results and effect sizes are given in the tables.

### Relations Between Autism Spectrum Disorder Symptomatology and Study Variables

Our first research question explored the relations between ASD symptomatology and alexithymia, trait EI and college adjustment through Pearson correlations tests. In terms of the associations between ASD symptomatology and alexithymia, higher SRS-2 raw scores were related to higher alexithymia total scores on the TAS-20, *r*(144) = 0.78, *p* < 0.001. Additionally, higher scores on the SRS-2 were associated with higher scores on each of the subscales of the TAS-20. This included difficulty identifying feelings (DIF), *r*(149) = 0.74, *p* < 0.001, difficulty describing feelings (DDF), *r*(149) = 0.68, *p* < 0.001, and externally oriented thinking (EOT), *r*(146) = 0.50, *p* < 0.001. As shown in Table 2, the same pattern of findings held for the both DSM-5 compatible subscales of the SRS-2 (i.e., *social communication and interaction* and *restricted interests and repetitive behaviors*). Lastly, higher ASD symptomatology scores were also related to elevated alexithymia scores that were at or above clinical threshold (≥61) for alexithymia, *r*(142) = 0.57, *p* < 0.001.

**TABLE 2:** Relations between study variables.

Variable	1	2	3	4	5	6	7	8	9	10	11
1. ASD symptomatology (SRS-2)	–										
2. SCI	0.99***	–									
3. RRB	0.84***	0.73***	–								
4. Total alexithymia (TAS)	0.78***	0.79***	0.59***	–							
5. DIF	0.74***	0.73***	0.60***	0.89***	–	–					
6. DDF	0.68***	0.69***	0.51***	0.88***	0.74***	–					
7. EOT	0.50***	0.51***	0.35***	0.73***	0.41***	0.47***	–				
8. Trait EI (TEIQue-SF)	–0.72***	–0.72***	–0.54***	–0.68***	–0.67***	–0.61***	–0.38***	–			
9. Academic adjustment	0.056	0.08	–0.02	0.06	0.04	0.09	0.05	–0.08	–		
10. Social adjustment	–0.28***	–0.30***	–0.15	–0.20*	–0.20**	–0.13	–0.13	0.36***	0.47***	–	
11. Personal-emotional adjustment	–0.31***	–0.29***	–0.31***	–0.30***	–0.32***	–0.22**	–0.19*	0.34***	0.53***	0.18*	–

SRS-2, Social Responsiveness Scale-2; SCI, Social Communication and Interaction subscale; RRB, Restricted Interests and Repetitive Behavior subscale; TAS, Toronto Alexithymia Scale; DIF, Difficulty Identifying Feelings subscale; DDF, Difficulty Describing Feelings subscale; EOT, Externally Oriented Thinking subscale; TEIQue-SF, Trait Emotional Intelligence Questionnaire-Short Form. *p < 0.05, **p < 0.01, ***p < 0.001.

Additionally, ASD symptomatology was related to lower trait EI scores, *r*(149) = –0.72, *p* < 0.001. As shown in Table 2, the two dimensional subscales of the SRS-2 were each significantly related to lower trait EI. Finally, significant correlations were also found between ASD symptomatology and social adjustment, *r*(141) = –0.28, *p* < 0.001, and personal-emotional adjustment to college subscales, *r*(142) = –0.31, *p* < 0.001, on the SACQ. However, ASD symptomatology was not significantly related to academic adjustment, *r*(141) = 0.06, *p* = 0.508.

Our second set of research questions examined the associations between alexithymia and trait EI and alexithymia and college adjustment. As expected, a significant negative relation was found between alexithymia and trait EI, *r*(143) = –0.68, *p* < 0.001. This pattern extended to each of the subscales of the TAS-20, which were also negatively associated with trait EI (see Table 2). Although we were not sure whether alexithymia would be related to college adjustment, significant negative relations were found between alexithymia and social adjustment, *r*(135) = –0.20, *p* < 0.001, and personal-emotional adjustment, *r*(136) = –0.30, *p* < 0.001, to college. However, alexithymia was not related to academic adjustment, *r*(135) = 0.06, *p* = 0.458 (see Table 3).

**TABLE 3:** Regression results for research questions.

Predictors of trait emotional intelligence (TEIQue-SF) Overall model *F*(2, 142) = 87.77, *p* < 0.001; Adjusted *R*^2^ = 0.55					
Variable	*B*	Beta	*t*	Sig	VIF
ASD (SRS-2)	–0.454	–0.484	–5.40	*p* < 0.001	2.53
Alexithymia (TAS-20)	–0.560	–0.305	–3.40	*p* < 0.001	2.53

**Predictors of social adjustment in college (SACQ)** **Overall model *F*(3, 134) = 7.73, *p* < 0.001; Adjusted *R*^2^ = 0.13**					
**Variable**	***B***	**Beta**	***t***	**Sig**	**VIF**

ASD (SRS-2)	–0.032	–0.152	–1.07	*p* = 0.285	3.09
Alexithymia (TAS-20)	–0.087	–0.211	–1.53	*p* = 0.128	2.91
EI (TEIQue-SF)	–0.094	0.406	3.23	*p* = *0.002*	2.44

**Predictors of personal-emotional adjustment in college (SACQ)** **Overall model *F*(3, 134) = 6.61, *p* < 0.001; Adjusted *R*^2^ = 0.11**					
**Variable**	***B***	**Beta**	***t***	**Sig**	**VIF**

ASD (SRS-2)	–0.050	–0.144	–1.01	*p* = 0.315	3.09
Alexithymia (TAS-20)	–0.041	–0.060	–0.444	*p* = 0.658	2.77
EI (TEIQue-SF)	0.072	0.193	1.59	*p* = 0.113	2.23

*SRS-2, Social Responsiveness Scale (Adult; Constantino and Gruber, 2012); TAS-20, Toronto Alexithymia Scale (Bagby et al., 1994); TEIQue-SF, Trait Emotional Intelligence Test (Petrides, 2009); SACQ, Student Adaptation to College Questionnaire subscales-revised (Baker and Siryk, 1999); VIF, Variance Inflation Factor; standard rule = values = 5 reflect multicollinearity issues (Gareth et al., 2013).*

### Predictors of Trait Emotion Intelligence and College Adjustment

Given these significant patterns of correlations, our third research question explored whether ASD symptomatology or alexithymia was a better predictor of trait EI. Simultaneous regression analyses showed that the overall model was significant, *F*(2, 142) = 87.77, *p* < 0.001, adjusted *R*^2^ = 0.55. Moreover, both alexithymia and ASD symptomatology were found to be significant predictors of trait EI. These results are shown in Table 3.

To address our final research question, we used simultaneous regression analyses to explore the best predictors of academic, social and personal-emotional adjustment to college in students. Predictors included alexithymia, ASD symptomatology and trait EI total scores. The analyses showed that the overall models were significant for social adjustment to college, *F*(3, 131) = 7.73, *p* < 0.001, adjusted *R*^2^ = 0.13, and personal-emotional adjustment, *F*(3, 131) = 6.61, *p* < 0.001; adjusted *R*^2^ = 0.11. However, the overall model for academic adjustment was not significant, *F*(3, 131) = 0.311, *p* < 0.817, adjusted *R*^2^ = 0.007. Our results show that only trait EI was a significant predictor of social adjustment (β = 0.406, *p* = 0.002), whereas alexithymia and ASD symptomatology were not significant predictors of academic, social or personal-emotional adjustment to college. These results are shown in Table 2.

## Discussion

The findings of the present study are consistent with past studies (e.g., Hill et al., 2004; Berthoz and Hill, 2005), with greater levels of ASD symptomatology associated with significantly higher total and subscale alexithymia scores. Additionally, higher ASD symptomatology scores were also related to elevated scores that were at or above clinical threshold (≥61) for alexithymia. These findings are important because the presence of co-occurring ASD symptomatology and alexithymia increases the chances of mood disorders and other psychiatric conditions that impinge upon both socio-emotional and general functioning (Grabe et al., 2008; Kinnaird et al., 2019).

In fact, it has been asserted that it is alexithymia and not autism *per se*, that contributes to emotion impairments (Bird and Cook, 2013). Support for this assertion has been found on different emotion recognition tasks (Kätsyri et al., 2008; Cook et al., 2013). In the present study, the connections between alexithymia and trait emotional intelligence (trait EI) were explored because trait EI reflects a set of important emotional competencies, including social awareness, emotion perception, and emotion regulation (Petrides et al., 2007b). It has also been shown to have significant associations with self-monitoring and empathic perspective (Schutte et al., 2001), measures of psychological adjustment (Chapman and Hayslip, 2005) and social network quality and life satisfaction (Laborde et al., 2014; Andrei et al., 2015).

In the present study, higher levels of alexithymia were related to lower trait EI. However, higher ASD symptomatology was also related to lower trait EI. This finding is consistent with those of Gökçen et al. (2014), who showed that higher scores on the Autism Quotient scale were negatively correlated with global trait EI as well as wellbeing, emotionality, sociability and empathy factors on the trait EI measure. This raised the question about whether alexithymia or ASD symptomatology served as a better predictor of trait EI. In our regression model, it was found that each were significant predictors of trait EI. These results suggest that alexithymia and ASD symptomatology both contribute to trait EI, which would be consistent with the fact that not all individuals with ASD exhibit elevated levels of alexithymia whereas social-emotional difficulties are common in ASD (American Psychiatric Association [APA], 2013). However, because trait EI encompasses a range of emotion processing abilities, it will be important for future studies to delineate the unique contributions of ASD symptomatology and alexithymia on specific emotion skills.

Finally, while scores on emotion measures may be important, they do not necessarily reflect everyday adjustment or functioning. Thus, our final research question explored whether alexithymia, ASD symptomatology and trait EI were significant predictors of adjustment to college. Previous research has shown that adjustment to college can take different forms, including academic (e.g., how well the student manages the academic demands), social (e.g., the degree to which the student has integrated themselves into the social milieu of college) and personal-emotional (e.g., students’ psychological and physical wellbeing) adjustment (Credé and Niehorster, 2012). Although significant correlations were found between adjustment to college and study variables, including alexithymia and ASD symptomatology, of the predictors assessed (i.e., alexithymia, ASD symptomatology and trait EI), only trait EI was a significant predictor and only for social adjustment. Thus, study findings provide evidence of the potential role trait EI may play in college adjustment, particularly in terms of a student’s integration into the social milieu of college. This assertion is consistent with previous studies showing that in the general population, trait EI is associated with overall wellbeing and social-personal success (e.g., Petrides et al., 2007b; Andrei et al., 2015).

Our results are also noteworthy in terms of their implications. In the general population, trait EI can be developed through intervention and training programs, producing beneficial outcomes in different settings (e.g., work, school, relationships; see Kotsou et al., 2019, for a review). Thus, future studies of trait EI are needed, including those that examine the potentially beneficial outcomes of training programs in the college setting, especially in students who show co-occurring ASD symptomatology and alexithymia. Exploring the impact of trait EI training programs on alexithymia would also be valuable.

### Limitations and Future Directions

Although we feel our results are compelling, limitations in the research must be acknowledged. First, self-report measures of alexithymia, such as the TAS-20, assume that individuals with alexithymia can accurately gauge their emotion abilities (Lane et al., 1996; Lumley et al., 2007). Although this is a legitimate concern, studies have shown significant associations between self-report measures of alexithymia and observational measures, as well as parent/other reports (e.g., Kooiman et al., 2002; Milosavljevic et al., 2016; Maroti et al., 2018). Others have shown that there are cognitive and affective components to alexithymia that are not captured by the TAS but may have bearing on college performance (Ziermans et al., 2019). Additionally, ASD symptomatology was determined based on a self-report measure and therefore likely represents a “high-functioning” group, limiting the generalizability of the findings. Nevertheless, Frazier et al. (2014) found that the SRS-2 exhibited measurement invariance across age, sex, and reporter (self vs. others). Others have used this sampling strategy to include individuals that may not have received a formal diagnosis of ASD but score above threshold on one or more self-report measures of ASD symptomatology (e.g., Trevisan and Birmingham, 2016; Dijkhuis et al., 2020; Lei et al., 2020). It has also been suggested that the SRS-2 may be capturing symptoms of other conditions, such as elevated levels of anxiety (South et al., 2017). We would concur and acknowledge that it can be difficult to tease apart co-occurring conditions with ASD. Thus, these findings do not preclude the possibility that other factors could be better predictors of trait EI and adjustment to college.

It must also be acknowledged that a different pattern of results could have been found if we had limited the study to only those with a formal diagnosis of ASD. In fact, it has been asserted that individuals with a formal diagnosis of ASD *and* are at or above threshold for alexithymia are a distinct subgroup, and that identification of that subgroup may have important implications for accurately determining the role of alexithymia in ASD (Kinnaird et al., 2019). Based on our findings, we would add that identifying this subgroup may be important for determining who may benefit the most by training programs of trait EI.

Nevertheless, our findings revealed that neither ASD symptomatology nor alexithymia predicted students’ adjustment to college. Rather, the only significant predictor of college adjustment was trait EI. These findings may reflect the fact that high-functioning individuals with ASD, or elevated levels of alexithymia, are not necessarily at a disadvantage in the college setting. With that said, programs that develop trait EI may also have a positive impact on alexithymia. Thus, college support programs that focus on developing trait EI may be valuable resource for students with and without ASD symptomatology.

## Data Availability Statement

The raw data supporting the conclusions of this article will be made available by the authors, without undue reservation.

## Ethics Statement

The studies involving human participants were reviewed and approved by the Institutional Review Board at Loyola University Chicago. The patients/participants provided their written informed consent to participate in this study.

## Author Contributions

DD designed and executed the study, analyzed the data, and wrote up the results. DM assisted with the data analyses and write-up of the study. Both authors contributed to the article and approved the submitted version.

## Conflict of Interest

The authors declare that the research was conducted in the absence of any commercial or financial relationships that could be construed as a potential conflict of interest.

## Publisher’s Note

All claims expressed in this article are solely those of the authors and do not necessarily represent those of their affiliated organizations, or those of the publisher, the editors and the reviewers. Any product that may be evaluated in this article, or claim that may be made by its manufacturer, is not guaranteed or endorsed by the publisher.
